# Preparation of Si-Ca-Fe Ceramsite from Multiple Solid Wastes for Cd(II) Removal: Adsorption Performance and Mechanism

**DOI:** 10.3390/ma19153253

**Published:** 2026-08-01

**Authors:** Dejian Pei, Shaoguang Hua, Feng Jiang, Anqi Zhu

**Affiliations:** 1 State Key Laboratory of Metal Mining Safety and Disaster Prevention and Control, National Engineering Research Center for Efficient Recycling of Metallic Mineral Resources, Sinosteel Maanshan General Institute of Mining Research Co., Ltd., Ma’anshan 243000, China; 2 School of Mechanical and Power Engineering, Nanjing Tech University, Nanjing 211816, China; 3 College of Life and Environmental Science, Central South University of Forestry & Technology, Changsha 410004, China

**Keywords:** Cd adsorption, novel ceramsite, solid waste, zero-valent iron

## Abstract

**Highlights:**

- The novel ceramsite for Cd removal is almost entirely (90 wt.%) from solid wastes;
- The phase evolution and adsorption performance of the novel ceramsite are analyzed;
- The ceramsite exhibits a notable Cd adsorption capacity, with a maximum value of 9.32 mg/g;
- The Cd adsorption behavior is governed by the ZVI in the ceramsite, coupled with its fine pores.

**Abstract:**

The increasing accumulation of industrial solid waste and worsening groundwater pollution pose significant environmental challenges. This study introduces a Si-Ca-Fe-based ceramsite from solid wastes with exceptional Cd adsorption capacity. A comprehensive investigation was conducted on the phase evolution, adsorption properties, and underlying mechanisms of the synthesized ceramsite. The findings revealed that the optimum sintering temperature for the ceramsite, characterized by austenite and pyroxene, was 1140 °C, which balanced mechanical strength and Cd adsorption capacity. Remarkably, the ceramsite (6.0 g) was immersed in 5 L of a Cd(NO_3_)_2_ solution for 21 h under initial conditions (pH = 7 and temperature = 20 °C), and the ceramsite exhibited a notable Cd adsorption capacity of 5.47 mg/g (initial Cd concentration: 53.42 mg/L), with a maximum theoretical capacity of 9.32 mg/g according to the Langmuir isotherm. An in-depth analysis of adsorption kinetics, phase composition, and EDS data indicated that the primary adsorption mechanism was the zero-valent iron (ZVI) corrosion-driven reaction. This ZVI formed in situ under reducing conditions during the ceramsite’s preparation and subsequently aided in the precipitation of Cd(OH)_2_. Additionally, the honeycomb structure of the ceramsite, containing fine pores (approximately 2–5 μm), enhanced physical adsorption via capillary action, further improving Cd removal. These insights offer a robust foundation for crafting efficient, solid waste-derived ceramsite tailored for heavy metal extraction from polluted water, presenting a compelling approach to concurrent waste recycling and environmental remediation.

## 1. Introduction

China has been the world’s largest producer and consumer of metals for 18 years, currently accounting for over 40% of global metal production [1]. This predominance is accompanied by extensive mining and smelting activities that have led to significant environmental impacts, particularly concerning industrial solid waste. In 2022, the metal industry contributed to 41.7% of China’s total industrial solid waste, amounting to approximately 1.71 billion tons [2]. Despite growing awareness and policy support for comprehensive utilization of this waste, initiatives in China started relatively late. As a result, the cumulative stockpile of bulk solid waste has reached an estimated 60 billion tons, with an annual increase of nearly 3 billion tons. Common types of this waste include iron tailings, steel slag, and coal gangue, which are typically characterized by low utilization rates, large storage volumes, and limited economic value [3,4,5]. Additionally, river sludge has emerged as a critical issue in urban sustainable development due to its accumulation in rivers during urbanization, posing challenges for both ecological systems and urban management [6,7].

Simultaneously, China’s rapid industrial expansion—especially in mining, smelting, and chemical manufacturing—has proceeded with only limited awareness of or regard for subsurface environmental protection. Consequently, large tracts of the country’s shallow and middle-depth aquifers are now contaminated by priority heavy metals such as Cr(VI), Cd, Pb, Hg, Ni, and Zn. Research over the past decade has therefore focused on replacing costly commercial resins or zero-valent iron (ZVI) with waste-derived or naturally abundant alternatives—e.g., biochar produced from agricultural residues, mine tailings-based zeolites, red mud-originating layered double hydroxides, sulfur-modified apatite, and sulfidated or phosphate-stabilized steel slags [8,9,10,11,12].

Extensive research has been conducted on the preparation of adsorption ceramsite using typical solid wastes. Wang et al. employed steel slag, carbon, and clay as primary raw materials to fabricate ceramsite capable of adsorbing lead ions from wastewater [13]. When the ratio of raw materials was 4:4:2, the firing temperature was 800 °C, and the pH value of the solution was between 6 and 10, the prepared ceramsite had the best adsorption effect. Liu et al. developed a steel slag-based ceramsite by combining steel slag, clay, and flour for phosphorus removal in packed columns [14]. The results showed that when the contact time was 4 h, the filter made from this ceramsite could remove 7.9 mg/g of phosphorus within 140 days under an influent concentration of 10 mg/L. Gao Xiang et al. investigated the metakaolinization temperature of coal gangue as the main raw material and prepared functional ceramsite loaded with chitosan for removing Cr(VI) ions from simulated heavy metal wastewater [15]. The results showed that when the calcination time was 60 min at 800 °C, the activity of the coal gangue was the highest, and the ceramsite prepared by mixing calcined coal gangue and chitosan according to a mass ratio of 1:15 had the highest Cr(VI) removal rate of 75.6%. It is worth noting that the synergistic use of multiple typical solid wastes to prepare ceramsite can not only break through the limitations of single components, realizing the massive addition of solid wastes, but also obtain better adsorption effects, which has gradually become a research hotspot. However, there are still two key scientific problems in the current research: (1) There is a lack of systematic research on the physical and chemical properties and structural characteristics of typical solid wastes, as well as the component reconstruction mechanism generated by the synergistic reaction of multiple solid wastes, which makes it impossible to establish an efficient supporting system. (2) The firing mechanism during the preparation of ceramsite and the corresponding heavy metal adsorption mechanism remain unclear.

To address the escalating issues of solid waste accumulation and groundwater contamination by toxic heavy metals, an innovative Si–Ca–Fe ternary ceramsite primarily synthesized from industrial and urban residues, including iron tailings, steel slag, coal gangue, and river sludge, was developed in this work. The developed ceramsite demonstrates exceptional adsorption efficiency and economic feasibility. A comprehensive examination of its phase evolution, adsorption behaviors and mechanisms towards Cd was conducted, laying a robust scientific foundation for employing solid waste-derived materials in combating heavy metal pollution. By endorsing circular economy principles, this research offers empirical evidence supporting the application of solid waste-based Si–Ca–Fe ceramsite in permeable reactive barriers and analogous heavy metal removal contexts. Consequently, it bridges the gap between theoretical concepts and their practical deployment in sustainable groundwater remediation efforts.

## 2. Experiments and Methods

### 2.1. Raw Materials

In this study, four solid wastes, namely iron tailings, steel slag, coal gangue, and river sludge, were collected from mines, steel enterprises, and the Cihu river channel in Maanshan, China, with the aim of utilizing these materials as raw materials for the preparation of ceramsite. The chemical composition of these wastes is detailed in Table 1. As can be seen, iron tailings and coal gangue are predominantly composed of SiO_2_, Al_2_O_3_, and Fe_2_O_3_. Steel slag mainly consists of SiO_2_, CaO, and Fe_2_O_3_, while river sludge is primarily made up of SiO_2_ and Al_2_O_3_. The raw material proportion scheme for the ceramsite is shown in Table 2.

**Figure 1:**
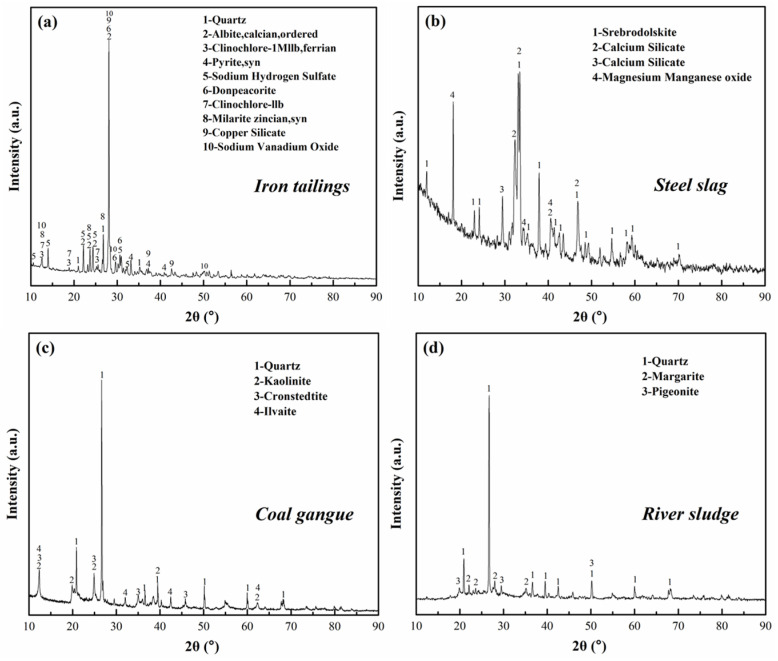
XRD patterns of raw materials: (**a**) iron tailings; (**b**) steel slag; (**c**) coal gangue; (**d**) river sludge.

Further analysis was conducted on the mineral compositions of these wastes, with the results depicted in Figure 1. The phases based on X-ray diffraction peak analysis are presented in Table 3. The mineral composition of iron tailings is relatively complex, encompassing Quartz (SiO_2_); Albite, calcian ordered ((Na, Ca)Al(Si, Al)_3_O_8_); Clinochlore-1Mllb, ferrian ((Mg, Fe, Al)_6_(Si, Al)_4_O_10_(OH)_8_); and Pyrite, syn (FeS_2_). The primary constituents of steel slag are Srebrodolskite (Ca_2_Fe_2_O_5_), calcium silicate (Ca_2_SiO_4_, Ca_3_SiO_5_), and magnesium manganese oxide (MgMnO_4_). Coal gangue mainly comprises Quartz (SiO_2_), Kaolinite (Al_4_(OH)_2_Si_4_O_10_), Cronstedtite (Fe_3_FeSiO_4_(OH)_5_), and Ilvaite (CaFe_3_Si_2_O_8_(OH)). River sludge primarily consists of Quartz (SiO_2_), Margarite (CaAl_2_(Si_2_Al_2_O_10_)(OH)_2_), and Pigeonite ((Fe, Mg, Ca)SiO_3_).

These solid wastes, which are typically considered by-products of industrial processes, have been identified as potential raw materials for ceramsite production due to their high contents of silica and alumina, which are essential components for the formation of ceramsite. The use of these wastes not only provides a sustainable solution for their disposal but also contributes to the conservation of natural resources.

### 2.2. Preparation Method

**Figure 2:**
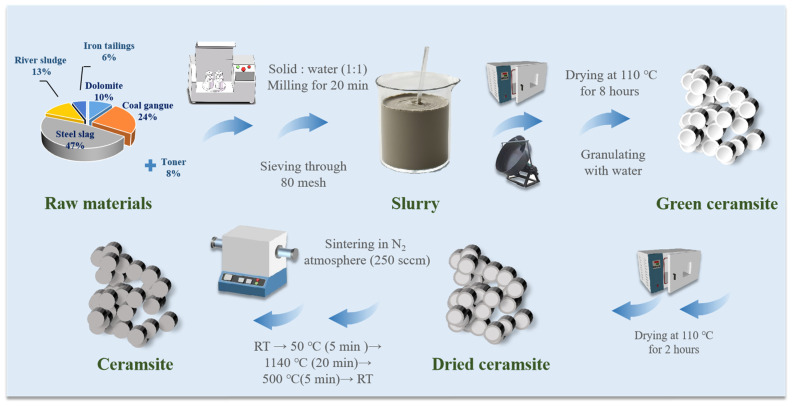
Preparation procedures for solid waste-based ceramsite.

The preparation process of solid waste-based ceramsite encompasses several critical steps, including mixing, milling, drying, granulating, and sintering, as illustrated in detail in Figure 2. Initially, the raw material powder is meticulously combined with water at a precise ratio of 1:1. This mixture is then subjected to wet ball milling for a duration of 20 min, utilizing a planetary ball mill to ensure thorough homogenization. The resulting slurry is sieved through an 80-mesh screen to remove any large particles and achieve a uniform consistency. Subsequently, the slurry is placed in a drying oven maintained at 110 °C for 8 h, a process that ensures complete dryness of the material. Once the powder is thoroughly dried, an appropriate amount of water is reintroduced to the mixture. This step is crucial as it adjusts the viscosity of the mixture to a level that is optimal for the preparation of green ceramsite. The green ceramsite, which is the preliminary form of the final product, is then placed in an oven at 110 °C for 2 h. This step further stabilizes the structure of the green ceramsite, preparing it for the final sintering process. The final stage involves sintering the dried green ceramsite in a tube furnace under a nitrogen (N_2_) atmosphere. The sintering process is carefully controlled to ensure the development of the desired properties in the ceramsite. The temperature is initially raised to 50 °C and held for 5 min. This initial heating phase is followed by a rapid increase in temperature to 1140 °C, where it is held for 20 min. The temperature is then gradually reduced to 500 °C and held for another 5 min before cooling to room temperature. Throughout this process, the heating and cooling rates are meticulously maintained at 10 °C per minute. This controlled sintering process ensures the formation of a ceramsite with desirable physical and chemical properties.

### 2.3. Characterization

The chemical composition of the samples was meticulously analyzed using an X-ray fluorescence spectrometer (ZSX Primus II, Rigaku, Akishima, Japan), which is renowned for its high precision and ability to detect a wide range of elements. The mineralogical phases were identified by X-ray diffraction (XRD) (Ultima IV, Rigaku, Akishima, Japan). This technique is highly effective in determining the crystalline structure and composition of materials. The microstructure and elemental distribution were examined using scanning electron microscopy with energy-dispersive spectroscopy (SEM-EDS) (SU8020, Hitachi, Tokyo, Japan). This method provides detailed information on the morphology and elemental composition at the microscopic level. Cd concentrations were measured via inductively coupled plasma optical emission spectrometry (Optima 8000, PerkinElmer, Waltham, MA, USA), which is a highly sensitive technique for determining trace elements.

The crushing and wear rate of the ceramsite was referenced to the standard CJ/T 299-2008 [16], C  =  m_b_/m × 100%, in which C is the crushing and wear rate of ceramsite, m_b_ represents the mass of sample less than 0.5 mm sieve, and m denotes the total mass of the ceramsite sample.

In the adsorption experiments, each portion of ceramsite (6 ± 0.2 g) was immersed in 5 L of a Cd(NO_3_)_2_ solution under initial conditions (pH = 7 and temperature = 20 °C). For the kinetic study, the initial Cd concentration was set at 10.25 mg/L, and 5 mL aliquots were extracted at various time intervals (1–21 h) to monitor the adsorption process over time. For the isotherm study, the initial Cd concentrations were varied (10.25, 53.42, 151.57, 249.75, and 331.79 mg/L), and 5 mL aliquots were extracted after 21 h of adsorption to evaluate the adsorption capacity at different concentration levels. The adsorption experiments were performed in duplicate for each group.

The formula for calculating adsorption capacity is (C_0_-C) × 5/6 (mg/g). The description of the calculation of q_e_ is (C_0_-C_e_) × 5/6 (mg/g). The description of the calculation of the removal rate is [(C_0_-C)/C_0_] × 100% (%). C_0_ is the initial concentration, C is the concentration during adsorption, and C_e_ is the concentration at adsorption equilibrium.

## 3. Results and Discussion

### 3.1. Phase Evolution of Si-Ca-Fe-System Ceramsite

**Figure 3:**
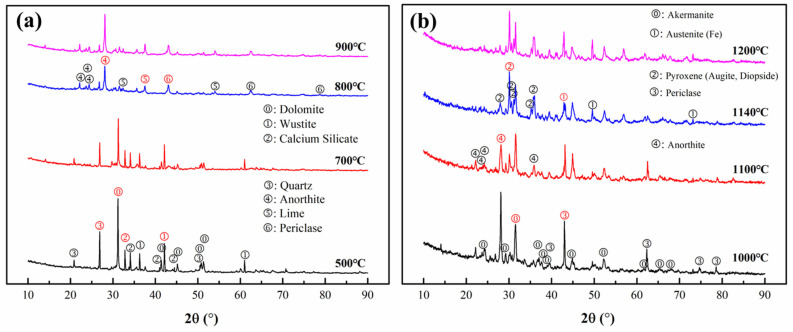
Phase analysis of Si-Ca-Fe ceramsite at different sintering temperatures (**a**) 500–900 °C; (**b**) 1000–1200 °C.

**Figure 4:**
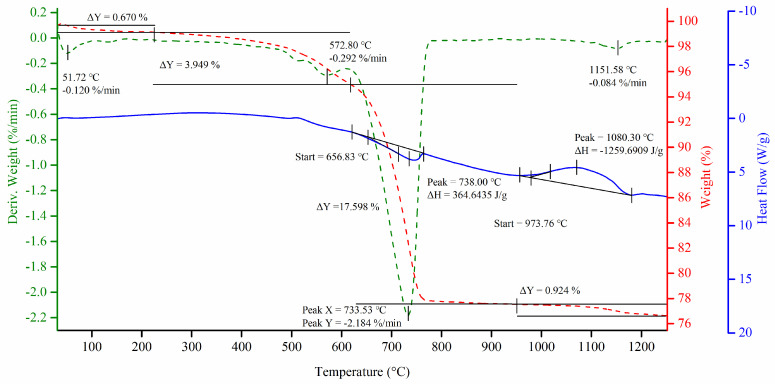
DSC-TG analysis of solid waste-based ceramsite green body in Si-Ca-Fe system.

The mineral phase composition is a direct determinant of ceramsite performance. This study investigated the alterations in mineral phase composition (Figure 3) and its corresponding DSC-TG curves (Figure 4) throughout the ceramsite sintering process, shedding light on the evolutionary trajectories of these mineral phases. The findings indicate that at a sintering temperature of 500 °C, the predominant mineral phases in the ceramsite samples mirror those found in the raw materials. These include Dolomite (CaMg(CO_3_)_2_) and Wustite (FexO) from steel slag and calcium silicate (Ca_2_SiO_4_) and Quartz (SiO_2_) derived from coal gangue and river sludge. Certain components undergo mass reduction due to processes like volatilization and dehydration. Upon heating to 800 °C, Dolomite undergoes complete decomposition, characterized by a marked endothermic reaction yielding Lime (f-CaO) and Periclase (f-MgO), alongside a substantial quantity of anorthite (CaAl_2_Si_2_O_8_). At 1000 °C, both the Periclase and anorthite concentrations peak, coinciding with the emergence of Akermanite (Ca_2_MgSi_2_O_7_) and trace amounts of austenite [PDF card: 52-0513]. Further elevation to 1100 °C witnesses a decline in anorthite levels, the initiation of pyroxene phase formation, and a zenith in Akermanite content. By 1140 °C, there is a continued rise in both pyroxene and austenite, with the former becoming the principal crystalline phase; simultaneously, the Akermanite phase wanes, accompanied by a distinct exothermic reaction. At 1151.58 °C, the onset of liquid phase generation facilitates densification. As the temperature escalates to 1200 °C, there is a marginal increase in both the austenite and gehlenite concentrations. It is speculated that during the iron oxide reduction process of the carbon powder material under a nitrogen atmosphere, the effects of nitrogen and elements such as Mn in the steel slag significantly expand the FCC-γ phase region and extend the temperature range of austenite existence down to room temperature, allowing austenite to remain stably present at room temperature. In essence, the fabrication and sintering procedures for Si-Ca-Fe-system solid waste-based ceramsite are distinguished by the decomposition, recombination, and regeneration of constituent elements—a stark departure from the conventional Si-Al system. Notably, the primary constituents of the Si-Ca-Fe system encompass SiO_2_ (44–55%) and CaO (13–28%), which can integrate intricate elements such as Fe, Mn, and Cr. During the sintering process, ceramic crystallization occurs prior to densification. This sequence facilitates precise temperature control and allows for adjustment of the pore structure, which might affect its adsorption performance.

**Figure 5:**
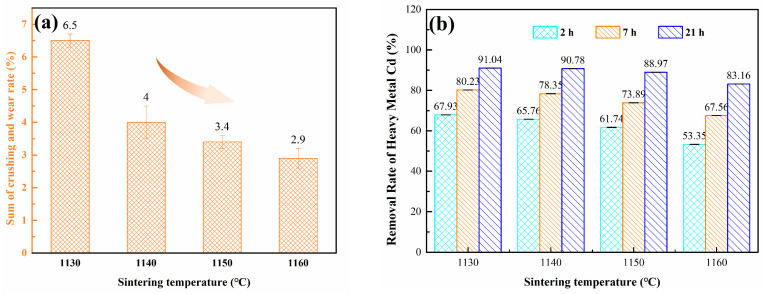
(**a**) Sum of crushing rate and wear rate; (**b**) removal rate of heavy metal Cd for ceramsite in Si-Ca-Fe system prepared at different sintering temperatures.

To further analyze the influence of the ceramsite densification process on its performance, this study investigated the mechanical properties (sum of breakage rate and abrasion value) and heavy metal Cd removal efficiency of ceramsite prepared at different sintering temperatures (1130 °C, 1140 °C, 1150 °C and 1160 °C), shown in Figure 5. The results showed that in the temperature range of 1130–1160 °C, with an increase in sintering temperature, the adsorption and removal capacity of ceramsite for Cd gradually decreased, while the mechanical properties were gradually improved. This may be due to the enhanced internal densification of ceramsite at higher temperatures, resulting in a reduction in pore structure. Among them, the sample sintered at 1130 °C had the best Cd removal ability; however, its mechanical properties did not meet the requirements (≤6%, CJ/T 299-2008). Therefore, the optimal sintering temperature for the Si-Ca-Fe-system ceramsite was determined to be 1140 °C. The toxicity leaching of ceramsite samples according to the Chinese standard (HJ/T 299 [17]) and the pH of the ceramsite samples were tested. The obtained heavy metals Mn, Cr, Ni, Zn, Pb, As, Cd, and Fe and the pH are shown in Table 4. Compared with the limit requirements of the standard (GB 8978 [18]), all data were low, except for the Fe concentration. The latter was not limited, and its leaching by acid resulted in a certain amount of dissolution that was related to the occurrence of Fe elements. Toxic heavy metals exhibited low leaching levels, mainly due to the phase reconstruction that occurred during the preparation process of ceramsite, especially the formation of the pyroxene phase.

### 3.2. Adsorption Characteristics of Si-Ca-Fe-System Ceramsite

**Figure 6:**
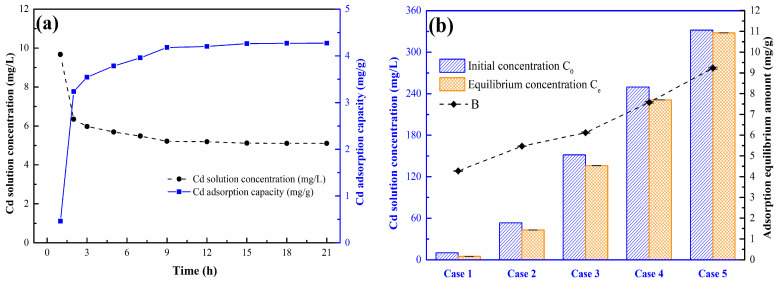

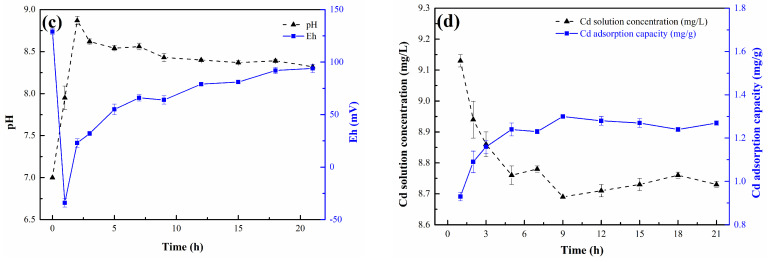
(**a**) Solution concentration and adsorption capacity of ceramsite containing ZVI after adsorption; (**b**) equilibrium concentration of Cd at different initial concentrations; (**c**) pH and Eh of the expanded clay adsorption process; (**d**) solution concentration and adsorption capacity of ceramsite prepared without carbon powder incorporation and nitrogen control after adsorption.

The adsorption properties of the prepared Si-Ca-Fe-system ceramsite were thoroughly investigated in this study. As depicted in Figure 6a, when the initial Cd concentration was set at 10.25 mg/L, the adsorption process exhibited a rapid increase within the first 3 h, after which the rate slowed down and eventually reached equilibrium at approximately 15 h. This initial rapid adsorption phase is indicative of a high affinity of the ceramsite for Cd^2+^, which is a desirable characteristic for efficient adsorption. The Fe^2+^ and Fe^3+^ concentrations were 8.62 mg/L and 8.15 mg/L respectively. After another 24 h, the Fe^2+^ and Fe^3+^ concentrations were 4.79 mg/L and 13.83 mg/L. The total content increased slightly, indicating that a small amount of Fe still reacted to form Fe^2+^ upon reaching adsorption equilibrium, but the Fe^2+^ was oxidized to Fe^3+^ due to the solution being exposed to air. This is consistent with the presence of hematite after the adsorption of ceramsite. The influence of different initial concentrations of Cd-containing solution on the adsorption performance was also examined, as illustrated in Figure 6b. It was observed that as the initial concentration of Cd gradually increased, the difference between the equilibrium concentration (C_e_) and the initial concentration (C_0_) progressively decreased. Concurrently, the amount of ceramsite adsorption at equilibrium gradually increased, demonstrating a positive correlation between the initial concentration and the adsorption equilibrium amount of ceramsite. This finding aligns with the general principle that higher initial concentrations provide a stronger driving force for adsorption, up to a certain limit. Notably, when the initial concentration was 53.42 mg/L, the prepared ceramsite exhibited a significant Cd adsorption capacity of 5.47 mg/g. This value surpassed that of iron tailings-based composites and steel slag–sludge-based ceramsite, which had capacities of less than 4 mg/g and 3 mg/g, respectively [19,20]. This superior adsorption capacity can be attributed to the unique composition and structure of the Si-Ca-Fe-system ceramsite, which provides a larger number of active sites for Cd adsorption. The pH and Eh during the adsorption process are shown in Figure 6c, and significant changes occurred in both during the early stage of adsorption. In the stage as adsorption reached equilibrium, the Eh increased due to dissolved oxygen, which is consistent with the changes in the adsorption process. A supplementary experiment was conducted starting from the preparation stage, where no carbon powder addition or nitrogen control was implemented in the preparation process. The adsorption process of the ceramsite is shown in Figure 6d and mainly occurred within 3 h. No Cd(OH)_2_ precipitate was observed during the experimental process, and the Cd removal rate was low. This further corroborates the important role of ZVI in the adsorption process.

**Figure 7:**
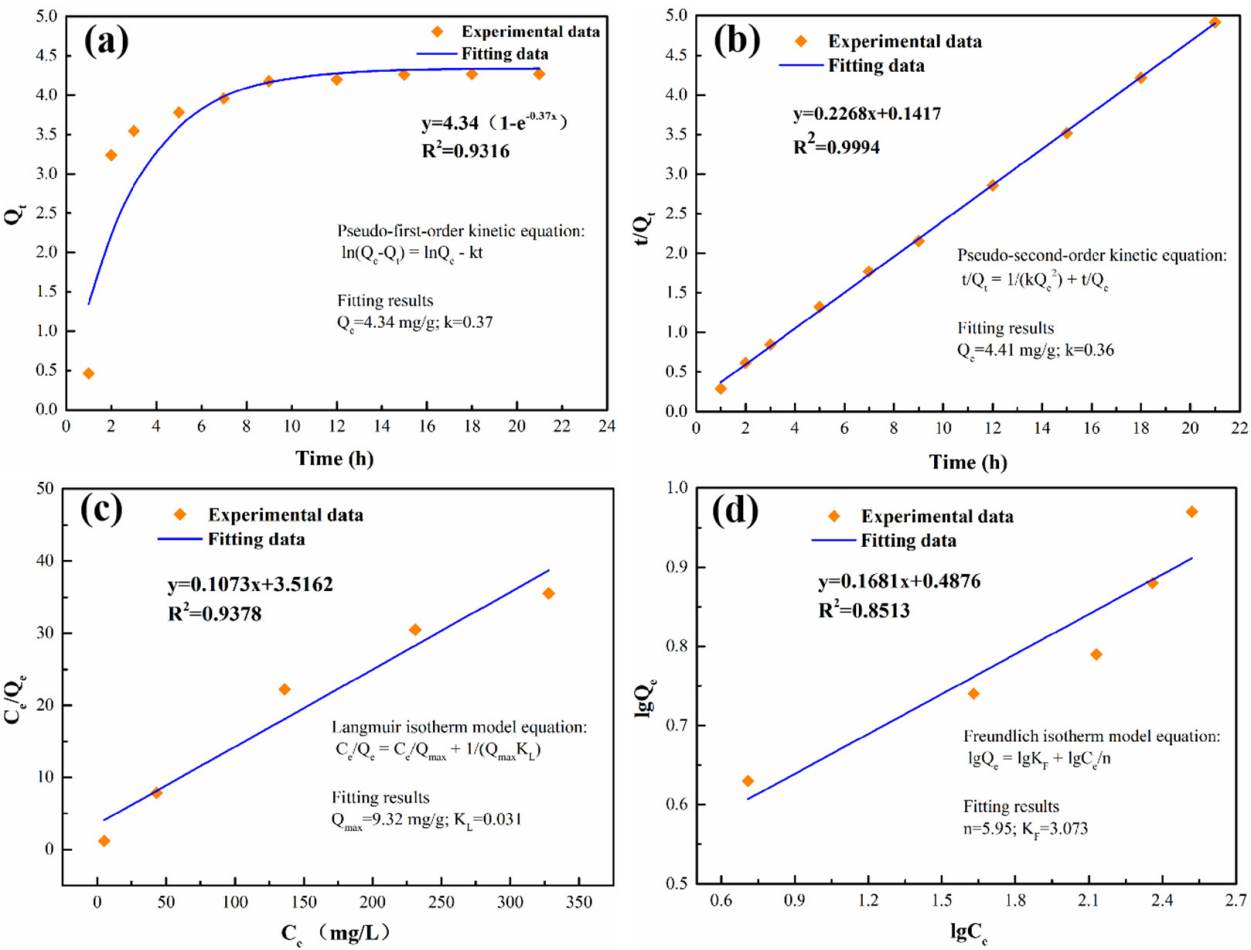
(**a**) Pseudo-first-order kinetic fitting; (**b**) pseudo-second-order kinetic fitting; (**c**) Langmuir isotherm model fitting; (**d**) Freundlich isotherm model fitting.

Furthermore, the kinetic characteristics and isotherm models of Cd adsorption by this ceramsite were studied, as shown in Figure 7. The adsorption process was found to conform more closely to the pseudo-second-order kinetic equation, with a correlation coefficient (R^2^) greater than 0.99. This suggests that the adsorption mechanism is likely controlled by chemical interactions, such as the formation of chemical bonds between Cd^2+^ and the functional groups on the ceramsite surface. In terms of isotherm modeling, the Langmuir model was found to be more suitable than the Freundlich model. The maximum adsorption capacity of the prepared ceramsite, as determined from the Langmuir model fitting, was 9.32 mg/g. The high maximum adsorption capacity suggests that the ceramsite is a promising material for the removal of Cd from contaminated water.

### 3.3. Adsorption Mechanism of Si-Ca-Fe-System Ceramsite

**Figure 8:**
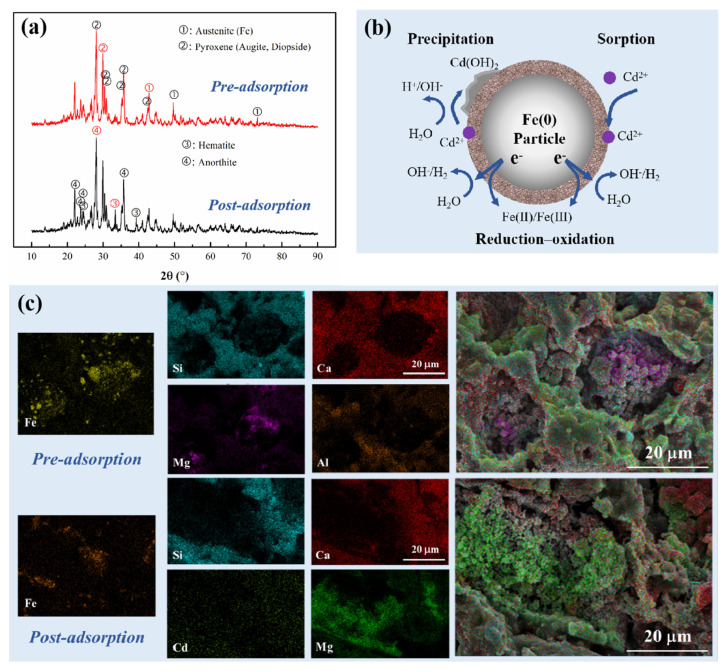
(**a**) XRD patterns of ceramsite pre- and post-adsorption; (**b**) schematic diagram of ZVI chemical adsorption process; (**c**) EDS analysis of ceramsite pre- and post-adsorption.

The adsorption mechanism of the ceramsite was thoroughly elucidated by examining the alterations in mineral phases before and after the adsorption process, as illustrated in Figure 8a. Prior to treatment, the sample was predominantly composed of pyroxene, which includes Augite and Diopside, anorthite, and austenite. These mineral phases provide a complex and reactive surface that facilitates the adsorption process. Post-treatment analysis revealed no notable alterations in the levels of pyroxene and anorthite within the sample. However, a marked reduction in ZVI and a pronounced increase in the hematite phase (Fe_2_O_3_) were evident. This transformation suggests that the ZVI in the ceramsite undergoes oxidation during the adsorption process, which is a critical step in the removal of Cd from the solution. By drawing upon the fitted curve of the pseudo-second-order kinetics, in conjunction with the adsorption kinetics model, it was deduced that the oxidation–reduction reaction involving ZVI in the ceramsite significantly contributes to the removal of Cd from the solution. This finding aligns with the general principle that redox reactions can enhance the adsorption capacity of materials by generating reactive sites that facilitate the binding of heavy metal ions. The relevant literature also reports that ZVI boasts potent redox potential and serves as an effective reducing agent. It facilitates the removal of heavy metals through mechanisms such as adsorption, precipitation, and encapsulation on the Fe surface, as described by Formulas (1)–(3) [21,22]. These mechanisms are further illustrated in Figure 8b, which provides a comprehensive understanding of the interaction between ceramsite and Cd ions for efficient adsorption. The combination of these mechanisms ensures that the ceramsite not only adsorbs Cd ions but also transforms them into a more stable form, thereby enhancing the overall effectiveness of the adsorption process.

Fe^0^ → Fe^2+^/Fe^3+^ + corrosion products (1)

≡Fe-OH + Cd^2+^ ⇌ ≡Fe-O-Cd^+^ + H^+^ (2)

Cd^2+^ + 2OH^−^ ⇌ Cd(OH)_2_ (s) (3)

**Figure 9:**
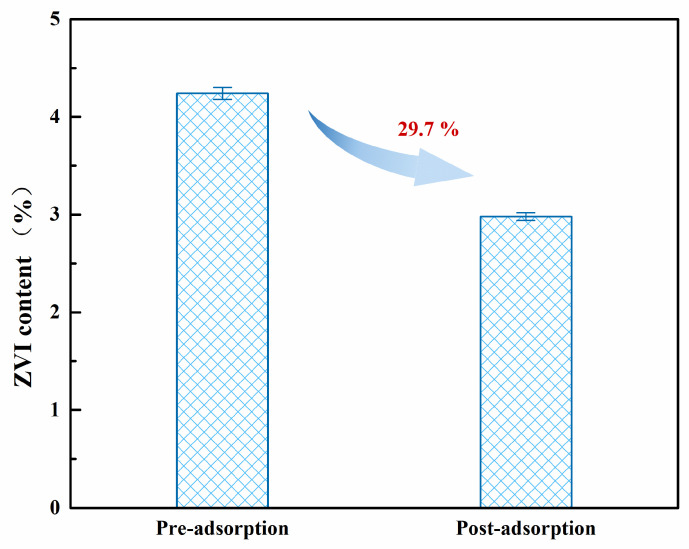
ZVI content of ceramsite pre- and post-adsorption.

The pre-treatment morphology of the ceramsite exhibited a distinct honeycomb-like structure, characterized by an extensive network of pores. These included both macropores and a significant number of fine pores, typically ranging within 2–5 μm in diameter. Such fine pores are capable of generating considerable capillary adsorption forces, which enhance the material’s capacity for the removal of heavy metal ions. SEM-EDS analysis conducted on the ceramsite prior to adsorption revealed the presence of iron in two distinct forms: relatively enriched iron-rich regions and more widely dispersed iron constituents. From this combined with the XRD results, it can be inferred that the enriched iron components correspond primarily to ZVI, whereas the dispersed iron is present within a solid solution phase, identified as pyroxene. Following the adsorption process, a notable reduction in the intensity of the enriched iron components was observed, as illustrated in Figure 8c. Quantitative analysis via chemical titration further confirmed this transformation, showing that the ZVI content decreased from 4.24% in the pre-adsorption sample to 2.98% post-adsorption (Figure 9). This marked reduction underscores the active participation of ZVI during the adsorption process. Therefore, the primary mechanism underpinning the adsorption of heavy metals in this system can be attributed to the chemical reactions involving ZVI—formed during the ceramsite preparation process—under reducing conditions, synergistically complemented by the physical adsorption effects facilitated by the fine porous structure.

## 4. Conclusions

This study successfully synthesized a high-efficiency and low-cost ceramsite for cadmium (Cd) adsorption, with nearly 90% of its raw materials derived from solid waste, highlighting its economic and environmental benefits. The adsorption characteristics of the as-prepared ceramsite were systematically investigated, and the underlying mechanisms were thoroughly elucidated. The main findings are summarized as follows:

(1) The sintering process of the Si-Ca-Fe-system ceramsite encompasses decomposition, recombination, and regeneration of mineral phases. The optimal sintering temperature was identified as 1140 °C, offering a balance wherein the ceramsite achieves the necessary mechanical strength while retaining commendable adsorption capacity for Cd.
(2) Under an initial Cd(II) concentration of 53.42 mg/L, the ceramsite exhibited a high adsorption capacity of 5.47 mg/g. The adsorption process was well described by the pseudo-second-order kinetic model and the Langmuir isotherm model, indicating that monolayer adsorption dominated and chemical interactions played a major role in Cd removal. Based on the Langmuir model, the maximum adsorption capacity was determined to be 9.32 mg/g.
(3) The decrease in ZVI content after adsorption, together with the appearance or intensification of iron oxide phases and Cd signals in SEM-EDS maps, suggests that ZVI oxidation and the formation of Fe-based surface precipitates contribute substantially to Cd(II) removal. Cd retention likely proceeds through a combination of surface complexation, adsorption or co-precipitation on Fe corrosion products, possible Cd(OH)_2_ precipitation under locally alkaline conditions, and physical retention within the porous ceramsite structure.

This study achieved the green recycling concept of “treating waste with waste” by successfully converting solid waste into high-performance ceramsite. This approach not only establishes a novel pathway for the harmless and high-value utilization of solid waste but also provides an efficient and low-cost solution for the deep purification of heavy metals in wastewater.

## Figures and Tables

**Table 1:** Chemical compositions of raw materials.

Chemical Compositions	SiO_2_	CaO	Al_2_O_3_	MgO	Fe_2_O_3_	SO_3_	Na_2_O	K_2_O	P_2_O_5_	MnO	LOI
Iron tailings	43.01	8.86	16.73	3.89	15.25	3.90	4.00	1.43	-	-	2.93
Steel slag	10.02	49.94	1.19	3.63	27.61	0.22	-	-	2.23	2.57	2.59
Coal gangue	50.86	3.03	24.88	0.73	13.13	3.92	-	-	0.11	0.19	3.15
River sludge	64.42	3.10	18.94	1.53	5.92	0.77	1.04	2.64	-	-	1.64

**Table 2:** Raw material proportion scheme for ceramsite.

Raw Material	Steel Slag	Coal Gangue	Iron Tailings	River Sludge	Dolomite	Toner
Proportion, %	47	24	6	13	10	8-external doping
Function performed	Main materials; providing Ca source, Fe source	Providing Si source; containing volatile substances conducive to reducing the sintering temperature	Providing Si source; alkaline components conducive to lowering the sintering temperature	Providing Si source; enhancing the plasticity of the green body	Providing Mg and Ca sources conducive to the formation of high-strength pyroxene phases	Acting as a reducing agent

**Table 3:** Mineral phase composition in different wastes.

Waste	Mineral Phase	Phase Composition	PDF Card
Iron tailings	Quartz	SiO_2_	79-1906
	Albite, calcian, ordered	(Na, Ca)Al(Si, Al)_3_O_8_	41-1480
	Clinochlore-1Mllb, ferrian	(Mg, Fe, Al)_6_(Si, Al)_4_O_10_(OH)_8_	07-0078
	Pyrite, syn	FeS_2_	71-2219
	Sodium hydrogen sulfate	Na_3_H(SO_4_)_2_	32-1090
	Donpeacorite	MgMnSi_2_O_6_	38-0358
	Clinochlore-IIb	(Mg_4.715_Al_0.694_Fe_0.269_Fe_0.109_Cr_0.128_Ni_0.011_) (Si_3.056_Al_0.944_)O_10_(OH)_8_	78-1997
	Milarite zincian, syn	KZn_3_Mn_2_Si_12_O_30_	72-1895
	Copper silicate	CuSiO_3_	70-3326
	Sodium vanadium oxide	Na_5_V_12_O_32_	24-1156
Steel slag	Srebrodolskite	Ca_2_Fe_2_O_5_	38-0408
	Calcium silicate	Ca_2_SiO_4_	23-1042
	Calcium silicate	Ca_3_SiO_5_	49-0442
	Magnesium manganese oxide	Mg_2_MnO_4_	19-0773
Coal gangue	Quartz	SiO_2_	46-1045
	Kaolinite	Al_4_(OH)_2_Si_4_O_10_	78-2109
	Cronstedtite	Fe_3_FeSiO_4_(OH)_5_	72-1673
	IIvaite	CaFe_3_Si_2_O_8_(OH)	78-1260
River sludge	Quartz	SiO_2_	89-1961
	Margarite	CaAl_2_(Si_2_Al_2_O_10_)(OH)_2_	18-0276
	Pigeonite	(Fe,Mg,Ca)SiO_3_	13-0421

**Table 4:** Statistical table of leaching toxicity (sulfuric acid and nitric acid method) and pH test results for ceramsite (mg/L).

Test Item	Test Result	Maximum Allowable Emission Concentration Standard Limit (GB 8978)
Mn	0.06	2.0
Zn	<0.006	2.0
Cd	<0.003	0.1
Pb	<0.05	1.0
Cr	<0.01	1.5
As	<0.0003	0.5
Ni	<0.01	1.0
Fe	647	/
pH	8.24	6–9

## Data Availability

The original contributions presented in this study are included in the article. Further inquiries can be directed to the corresponding author.
